# Childbirth in Early Swedish Television: From Promotion to Criticism of the Welfare State

**DOI:** 10.1007/s10912-025-09987-w

**Published:** 2025-12-13

**Authors:** Elisabet Björklund

**Affiliations:** Centre for Languages and Literature, Lund University, Box 201, Lund, 221 00 Sweden

**Keywords:** Childbirth, Television, Film, Swedish welfare state, Maternity care, Obstetrics

## Abstract

This article explores the shifting and competing ways in which childbirth, obstetrics, and maternity care were represented during the first two decades of television in Sweden. While childbirth on screen has a much longer history in both educational film and commercial cinema, the introduction of public service television in the late 1950s created a new space in Sweden for both educational and critical representations of reproduction, which had the potential of reaching a much larger national audience than was previously possible. Analyzing various television formats dealing with and displaying births from the early 1960s to the mid-1970s, this article examines how pregnant and birthing bodies were made visible in the new medium of television and what role these programs played in the larger debates on maternity care, obstetrics, and the Swedish welfare state in this period. Centrally, the article discusses the shift from a mode of representation in which childbirth was depicted within the framework of sex education or information about the welfare society’s support systems to feminist representations giving voice to women’s experiences and criticizing the medicalized perspective on childbirth found in Swedish healthcare. In this way, the article highlights shifting historical discourses of childbirth within the frames of a public service institution and a Nordic welfare state and emphasizes the importance of moving images as both an art form and an influential communication tool in postwar discussions of healthcare issues.

Undergoing an antenatal preparation class today often entails watching a film about childbirth. As Paula A. Michaels (2017) has observed, films showing births have become “a ubiquitous and indispensible [sic] component of antenatal classes, elevated to a rite of passage to parenthood” (24). For some expectant parents, this can be the first time they see an explicit depiction of birth. Others might already have watched several birth scenes on reality shows, YouTube, or Instagram, where the number of such scenes has multiplied in recent decades (e.g., Bliss 2020, 143–155; Bull 2016; Longhurst 2009; Tyler et al. 2013; Yam 2019). The widespread use of film in healthcare settings and the broad availability of birth films in the media constitute a contested visual culture of childbirth. While healthcare professionals are often concerned about the influence of information spread via commercial media, activists on social media are often driven by their commitment to diversifying media images and challenging dominant medicalized perceptions of childbirth (e.g., Yam 2019). Yet, these struggles over representation are not new to our present media culture. In fact, as many scholars have demonstrated, films and other media depictions of childbirth have circulated in various exhibition contexts since the early twentieth century. Moreover, questions of how birth should be represented, by whom, and in what media have been debated long before the advent of reality television and social media (e.g., Al-Gailani 2017; Eberwein 1999; Gainty 2017; Michaels 2017; Ostherr 2013; Parry 2013; Schaefer 1999; Strassfeld 2013). 

In this article, I explore one part of this longer visual history of childbirth by analyzing the shifting and competing ways of visualizing birth that emerged during the first two decades of television in Sweden, that is, from the late 1950s to the mid-1970s. This focus is relevant for three main reasons.

The first reason is that historical study of a specific national context can challenge generalized assumptions found in contemporary discourses around childbirth. As Sofia Bull (2016) has observed, much discussion of obstetric care and its depiction in the media today pits the “medical model” of childbirth, associated with advanced hospital care and the medicalization and pathologizing of birth, against the “natural birth model,” associated with minimal medical interventions, home births, and midwife-centered care. However, comparing American, British, and Scandinavian birthing shows on television, Bull (2016) demonstrated that British and Scandinavian programs present a more complex picture, often combining these two models. Hence, an exploration of how childbirth has historically been represented on Swedish television can provide contemporary debates on these issues with additional nuances.

The second reason is that the study of Swedish television can shed new light on the relationships among media, health, and medicine in the context of a Nordic welfare state. To this end, the 1950s, 1960s, and 1970s are an important period to study, as this was an era when the Swedish welfare state flourished (e.g., Schön 2012, 375–432). Issues related to sexuality and reproduction have long been important political concerns in Sweden, and during the decades following the end of World War II, they came to be strongly associated with Swedish welfare politics. The foundation for this image was laid in the 1930s, when the question of the declining birth rate became a major issue of debate in Sweden. This debate triggered many reforms in subsequent decades aimed at improving public health and living conditions for families, for example, different forms of economic support for expectant parents, free maternity and obstetric care, a general child allowance, and large-scale housing programs. Education in these issues was also strongly promoted. In 1955, Sweden introduced compulsory sex education in the schools, which became the starting point for forming Sweden’s international image as a country of sexual liberalism (e.g., Glover et al. 2009; Hale 2003; Lennerhed 1994). By the end of the 1960s, however, criticism of various aspects of the welfare state started to grow in Sweden. Studying media representations of childbirth from this period can reveal how this issue was perceived in relation to larger discussions of health and welfare.

The third reason is that studying early television can remind us that our time is not the only one when the media have been changing and had a great impact. By the end of the 1950s, when many of the reforms mentioned above were in place, television was introduced in Sweden and quickly became one of the most popular media (Höijer 1998; Weibull 2013). In fact, it was during the 1960s and 1970s that the impact of television was the most intense throughout the Western world. Unlike today, viewing choices were very few, as the number of television channels was limited; at the same time, television reached large parts of Western populations (von Hodenberg 2017, 75). This was clearly the case in Sweden, where a public service model had been chosen, with a monopoly held by the Swedish Broadcasting Corporation, and where there was only one channel until 1969 and only two from 1970 to the late 1980s. Thus, exploring how this influential new medium shaped the visual culture of pregnancy and childbirth can generate important knowledge of the relationship between media development and larger debates on healthcare issues; it can furthermore demonstrate how valuable visual material can be to historical research on childbirth and obstetrics.

I have two broader aims in this article. First, I aim to discuss how the different portrayals of childbirth appearing on television in the 1960s and 1970s related to changing views of the Swedish welfare state, in general, and to policies concerning maternity care and obstetrics, in particular. Second, I aim to explore how television as a new medium related to and influenced the larger visual culture around pregnancy and childbirth during this period.

## Method

To identify relevant programs for the study, I searched the digital database of the broadcasting company Sveriges Television (Sweden’s Television, SVT) during a visit to their department in Malmö, Sweden, and the online database of the audiovisual collections at the National Library of Sweden (Swedish Media Database). Here, I used a wide variety of search terms ranging from more general ones such as “pregnancy,” “birth,” and “reproduction” to more specific ones such as “cardiotocography” and “Cesarean section” (in Swedish). To complement these digital searches, I also browsed through all issues of *Röster i radio-tv* (Voices in radio-TV) between the years 1957 and 1975, a weekly magazine published by the Swedish Broadcasting Corporation, containing television schedules and articles about upcoming programs. Extant programs were accessed through the National Library of Sweden. For this article, I have limited my scope to television programs in which real births are captured on camera, which amounts to at least 17 programs during the period.^1^ This includes a variety of television formats, mostly documentary but also fiction (e.g., when a documentary birth scene is incorporated into a fictional framework). However, it excludes programs in which birth is staged, animated, or only a focus of discussion. The line is, however, sometimes difficult to draw, and the focus of my analysis is not only on the visualizations, but on whole programs—that is, the larger discourses or narratives around pregnancy and birth of which these scenes were part.

As the scope of the present article is limited, I have chosen to conduct a more in-depth analysis of a few programs, rather than discussing the entire corpus. Three programs are addressed here. First, I analyze an episode of *The face of Sweden: A series of self-portraits* (Bjelfvenstam 1962). This was a series of eight programs co-produced by the Swedish Institute for Cultural Exchange with Foreign Countries, the Swedish Broadcasting Corporation, and the National Educational Television and Radio Center (NETRC) of the USA. It targeted an American television audience and was partly funded by a large Swedish export promotion campaign in the USA in 1957, on the initiative of the Swedish Ministry of Trade, but it was also shown to Swedish television viewers in 1963 (Glover 2011, 98–107). My reason for highlighting this series is that its very first episode, “The secure society,” frames the issue of Swedish social security using the example of maternity and obstetric care, which makes it a good case for analyzing how pregnancy and childbirth were connected to welfare policies and Sweden’s international image during this era.

Second, I explore two feminist documentaries aired in the early 1970s. The first is entitled “‘…man blir ju kvinna…’ Om att få första barnet” (“‘…you become a woman…’ Having your first child”) and was the first episode of a series entitled *Livskris* (*Life crisis*, 1973), made by producers Monica Kempe, Noomi Liljefors, and Agnes Mannerheim. The second is a film entitled *Omställningen* (The change, 1974), made by left-wing documentary filmmaker Maj Wechselmann in collaboration with artist Anna Sjödahl, among others.^2^ The common aim of these programs was to create counter-images of the dominant representations of pregnancy and childbirth circulating during this era. Both explicitly criticized the medicalized nature of Swedish maternity and obstetric care, which was also tied to a larger criticism of modern Swedish society. I have chosen these programs as they also clearly connect the issue of giving birth to Swedish welfare policies, but from an entirely different perspective, which makes them fruitful to compare to *The face of* *Sweden*.

Despite this limited focus, the article also aims to connect the examples to wider discourses and representations of birth. To this end, my analytical approach is inspired by Kirsten Ostherr’s discussion of “medicine’s visual culture” in her book *Medical visions: Producing the patient through film, television, and imaging technologies* from 2013. Here, Ostherr employed the concept of visual culture to describe the heterogeneous landscape of medical images and visual media that has developed since the early twentieth century, including everything from medical imaging technologies, such as X-ray and ultrasound, to films used in public health campaigns and television hospital dramas (Ostherr 2013, 13–17). I adopt this perspective by not limiting my choice of material to a certain genre of television programs, but by instead trying to grasp the diversity of television formats in which birth scenes appeared during this period. Moreover, I relate my examples to the larger visual culture of birth at this specific historical moment by discussing how the programs built on and coexisted with a longer history of childbirth on film and other media depictions of birth.

The strength of this method is that it allows deep analysis of the meaning-making in a small group of television programs, while the historical contextualization and comparisons across genres and materials can demonstrate their wider significance. A weakness is that this case study approach is by definition very limited. Also, including a diversity of material could make the examples difficult to compare. The complexity of working with visual materials in general must also be acknowledged. For instance, an empirical study of the historical audience reception of these television programs is beyond the scope of this article. Employing terms developed by Gillian Rose (2016, 24–47), my approach is limited to mainly exploring these works at their “site of production” and at the “site of the image.” This means that I treat them as part of a technological change and in relation to conventions of depicting birth in previous and contemporary examples and media (i.e., the site of production) and by analyzing their mode of documentary representation using film analysis tools (i.e., the site of the image). However, my study cannot build knowledge of the various viewing contexts of these programs or how actual audiences understood them (i.e., the “site of circulation” and “site of audiencing”) (Rose 2016, 24–47).

This article is divided into three sections and a conclusion. In the first section, I describe childbirth on film before the introduction of television in Sweden in the 1950s, discussing the circulation and shifting function of birth films in various screening contexts during the first part of the twentieth century. This is followed by two analytical sections focused on the chosen examples. In the conclusion, I discuss the differences and similarities between the examples and the changes and continuities in society and the media they correspond to more widely.

## Childbirth on screen: Sex education, exploitation, and the avant-garde

Films showing documentary depictions of births existed long before the introduction of television. Examples can be found as far back as the era of silent film, and they have been shown in many different places and to various audiences. Films intended mainly for a medical audience were probably the earliest. Notably, American obstetrician Joseph DeLee recorded numerous births at his hospital in Chicago in the 1920s, and to do so, he even equipped one of his delivery rooms as a film studio (Gainty 2017). Around the same time, films showing births targeting a larger audience also began appearing. The best-known example is Soviet filmmaker Dziga Vertov’s classic experimental documentary *Chelovek s kino-apparatom* (*Man with a movie camera*, 1929), which contains an explicit depiction of a vaginal birth intercut with sequences from a funeral (Olszynko-Gryn et al. 2017; for another example, see Laukötter 2015).

However, in the first half of the twentieth century, birth films were often censored. In the USA, for instance, explicit depictions of pregnancy and birth were forbidden in films intended to be screened in mainstream cinemas, in line with the Production Code—the self-censorship guidelines agreed on by the major film companies that regulated film content from the early 1930s to the late 1950s (Kirby 2017). In Germany, the taboo of childbirth on film was not broken until the 1960s (Schwarz 2009, 205). In many countries, however, films with such content did find large audiences outside the mainstream cinema, not least in different types of sex education films. Many such films were made in collaboration with medical experts, organizations, or individuals engaged in sex education, which functioned to legitimize their controversial content and helped them pass censorship inspection (e.g., Björklund 2012, 47–94). Other films, however, belonged more clearly to a “low” type of culture. In the USA, the so-called exploitation film emerged in the late 1910s and existed alongside the classical Hollywood cinema until the late 1970s, dealing with topics that were not allowed in the mainstream cinema, such as sex, nudity, or drugs. The “sex hygiene film” was an important genre within this tradition, and many films of the kind attracted an audience by showing spectacular scenes of birth (Schaefer 1999).

The boundaries between sex education and exploitation, however, were often blurred. One well-known example of this is the American short film *The birth of a baby* (Christie 1938), which was produced with a clear educational aim, but also became a great success in commercial cinemas and was seen by five million people in the USA (Strassfeld 2013). Another phenomenon was that films could cross social boundaries when they crossed national borders. This, for example, happened with the exploitation film *Mom and dad* (Beaudine 1944), which contained a film-within-a-film showing both a vaginal birth and a Cesarean section when it was imported to Sweden in 1949. The film was cut by the censorship authority and adapted to the new market by a Swedish medical expert, who thus managed to give it a different status. The material used in films also circulated. Medical footage was sometimes reused in other films, and still images from films could appear in the press (Björklund 2024, 123–125). Images from *The birth of a baby* were, for instance, published in an issue of *Life* magazine, causing great controversy (Strassfeld 2013).

Birth films also crossed boundaries in another direction: from the educational to the avant-garde. At Amos Vogel’s film society Cinema 16 in New York, which screened films between 1947 and 1963, medical films were often shown, among them films depicting childbirth. Ostherr (2013) explained this with reference to the radical esthetic potential of birth scenes: “These images were triply taboo for being graphically ‘surgical,’ for exposing close-up views of female genitals, and for capturing and then exhibiting one of the most intimate, vulnerable moments in life for public display” (119).

Birth on film was thus clearly a controversial subject in the early twentieth century. Still, many people in both Europe and the USA came across films with such content. What these films showed and who had access to them, however, differed among national contexts. In Sweden, several films showing vaginal births were distributed commercially and even became quite popular in the 1940s and 1950s. Sweden’s censorship authority—the National Board of Film Censors—however, usually cut scenes of Cesarean sections, breech deliveries, or deliveries with forceps, as it was thought that these would frighten expectant mothers. Here, efforts to educate the public in matters related to sexuality and reproduction were paired with a distinction between “natural” and “pathological” birth and a paternalistic view of women, portraying them as vulnerable to visual impressions (Björklund 2012, 92–93; Björklund 2024).

In the 1950s and 1960s, when television had its breakthrough, new contexts for seeing birth films also appeared, partly due to new ideas about obstetric care. During the postwar period, hospital birth had increased dramatically to become the norm in most countries in the Western world. The high use of pharmacological pain relief that characterized this care gave rise to the natural birth movement, which spread through the parallel and competing methods of British obstetrician Grantly Dick-Read and French obstetrician Fernand Lamaze, who both saw great value in film (Al-Gailani 2017, 2018; Michaels 2014). In the following decades, numerous childbirth films were made for use in antenatal preparation classes, which was an expanding activity during the postwar decades (Michaels 2017). As earlier, information about reproduction also circulated via different media and exhibition contexts. For instance, Dick-Read’s film *Childbirth without fear* (1956) attracted immense attention when a clip from it was shown on the British television show *Panorama* in 1957 (Al-Gailani 2017).

It is difficult to determine to what extent films were used in Swedish antenatal preparation classes, but lists of educational material published in *Jordemodern*—the journal of the Swedish Association of Midwives—in the late 1960s and early 1970s indicate that several films were in circulation among midwives, at least six of which were birth films (Mjönes 1969, 388–389; Undervisningsmateriel (Undervisningsmateriel 1974), 287). The introduction of mandatory sex education in schools also meant an increase in films and other materials made for school use. In fact, most sex education films screened for schoolchildren in Sweden in the 1950s, 1960s, and 1970s focused on the biological process of reproduction, some of them ending with graphic depictions of childbirth (Björklund 2012, 281–287). Here, a few films based on the well-known pictures of fetal development taken by Swedish photographer Lennart Nilsson were among the most popular. In the 1960s, Nilsson’s photographs were published in the best-selling pregnancy advice book *Ett barn blir till* (*A child is born*, 1965), but they were also used in various sex education materials (Jülich 2011; 2015).

As we can see, in the late 1950s and early 1960s in Sweden, the visual culture of pregnancy was changing. On one hand, the sex education films shown in cinemas, in which audiences could see the process of (vaginal) birth on the big screen, were declining in popularity. This was partly because the introduction of sex education into schools meant that the market for these films was no longer as lucrative. But it was also related to the radicalization and liberalization of discussions of sex in the early 1960s, which began with criticism of the sex education taught in schools (Lennerhed 1994). A new generation of sex educators also appeared during these decades, and they shifted the focus to issues other than reproduction (Björklund 2012, 203–205). While opportunities to see birth on film in cinemas decreased during these years, information about pregnancy and birth multiplied in other media, one of which was television.

## Childbirth, education, and promotion of the Swedish welfare state

Regular television broadcasts began in Sweden in the latter part of 1956, which was late by international comparison. This was preceded by a public commission of inquiry, which in 1954 proposed that television in Sweden should be in the service of “society, culture, public education and homes” (quoted from Weibull 2013, 44) and that a monopoly should be held by the company Radiotjänst (Radio service), which already had the monopoly on radio broadcasts in Sweden. The government’s decision, which came in 1956, largely followed this proposal, which meant that Swedish television was organized as a public service institution and that Radiotjänst became the Swedish Broadcasting Corporation, with a monopoly on both radio and television and financed by license fees (rather than advertising). Newspapers and industry had formerly held shares in Radiotjänst, but the change meant that popular movements (e.g., trade unions, churches, and educational associations) also gained a substantial part of the ownership (40%). Lennart Weibull explained (2013, 44–45) that this was partly because the government wanted to demonstrate the public service character of the company. From its establishment, television in Sweden had a strong focus on education, as also reflected in the recruited personnel, most of whom were men with academic backgrounds who had previously worked in radio. “The academic and middle- to upper-class background of most of the television staff disclosed the kind of image that was desired—educational aspects (including those of a canonical, bourgeois taste and style) were indeed of the utmost importance,” wrote Tove Thorslund (2018, 42–43).

Television spread rapidly in Sweden. It took a few years before the broadcasts reached the whole country, but it soon became common to own a television set, and the number of licenses grew quickly. In 1963, 75% of households had a television, and this proportion would increase to 83% by the end of the decade. One must also consider that many people who did not own a television themselves visited friends and neighbors to watch the programs. In the early 1970s, practically, everyone had access to television (Höijer 1998, 156, 197–198, 216; Wachtmeister 1972, 141–142).

The introduction of television was also closely interwoven with developments connected to housing and domesticity. As Thorslund (2018) has explained, television was introduced in an era characterized by a strong political focus on the home. The “people’s home” had been, ever since the late 1920s, a central metaphor for the ideal society the Social Democrats wanted to create, and the baby boom of the 1940s contributed to a strong emphasis on families during the postwar decades. In the 1950s and 1960s, large-scale housing programs were initiated to solve problems of overcrowding and low living standards, and new suburban areas built especially for families were developed where people could work, live, and socialize (Thorslund 2018, 165–172; see also, e.g., Gråbacke et al. 2010). Thorslund (2018, 173–179) showed how the introduction of television influenced these new domestic spaces, as television sets needed to find a very concrete place within the home, as well as how related discourses fit well into an overall focus on the rationalization and standardization of the home during this period. Moreover, the 1950s was an era when most women still worked at home, meaning that many of the programs produced targeted this audience. For instance, a special production unit was formed that made programs about the home and family (Kleberg 1999; Thorslund 2018).

Television programs in which childbirth could be seen largely fit into two categories until the early 1970s: educational programs about reproduction and medical journalism programs dealing with technological advances in maternity care and obstetrics, such as new methods of pain relief during delivery. The largest proportion of programs belongs to the former category. Some of these were explicitly aimed at children, for example, a school television program entitled *Så kom du till* (How you were made) broadcast in 1966 and an episode of the series *Vill du veta* (Do you want to know) from 1971. Others were aimed at a general audience. The best-known example is the prize-winning television film *Så börjar livet* (*The beginning of life*, 1965), which narrated the course of pregnancy using Lennart Nilsson’s pictures and culminated in an explicit scene of the vaginal birth of a baby. The journalistic programs vary in their format. One early example is the documentary *Det första andetaget* (The first breath) from 1961, co-produced with the BBC, in which two births are shown briefly, while most of the program concerns techniques used to help newborns take their first breath. One of the earliest episodes of the medical program series *Ronden* (The round, 1966–1978), aired in 1966, reported on the new method of “pain-free” delivery, the paracervical block, and followed a woman giving birth to twins using this method. In other episodes of this series, births were shown as an introduction to discussions of other matters, such as prenatal diagnosis.

*The face of Sweden* does not clearly fit into any of these categories, in that it was produced to communicate an image of Sweden to American audiences. Still, it shares many narrative and stylistic devices with them and other educational films made in this period. For example, it clearly follows the documentary tradition that Bill Nichols (1991, 2017) has called “expository” documentary. This had been a dominant mode of documentary representation since the 1920s and was characterized by a so-called voice of God narrator (an invisible, often male, voice-over directly addressing the audience), supporting images, and editing serving a primarily rhetorical function (Nichols 1991, 34–38; Nichols 2017, 121–125). In an unpublished working paper, however, Malin Wahlberg discussed how the series also includes stylistic deviations from this mode, such as associative and ironic combinations of images, text, and commentary, as well as content with the potential to be provocative. One such example was the birth scene, which was considered too radical for an American audience, but which in the end was positively received in the USA (Wahlberg nd, see also Åhman 1963).

The first episode centers on the Swedish system of social security. This system developed in the decades following the end of World War II and is a tax-financed model largely characterized by universalism—the principle that social support such as retirement pensions, childcare benefits, and healthcare services should be available to everyone (e.g., Palme 2017). Many different aspects of this are introduced in the program, for example, healthcare more generally and care of the elderly, but the issue of maternity care and childbirth is used as a framework for the entire episode, as it begins with a woman receiving a positive pregnancy test result and ends with the delivery of her child. The issue is clearly used as the central example of Sweden’s “secure society.” Moreover, in contrast to many school films about reproduction from the period, this context entails that pregnancy and childbirth should primarily be dealt with as medical and social issues rather than biological ones. The purpose of introducing the viewers to the societal aspects of childbirth is made clear at the beginning of the film, when the speaker says:

It is now established principle that all women, married or not, should be well-prepared, physically and mentally, for childbirth. And that all should be encouraged to make full use of the wide range of free services available, from pregnancy tests, prenatal check-ups, and instruction courses to delivery itself.

The film uses the strategy of building a narrative around individuals to illustrate its points, featuring two Swedish women whom we follow through the film as they undergo preparation for childbirth. One woman’s pregnancy is progressing normally, while the other has a heart problem and needs special care. Nikolas Glover (2011, 94–98) called this device “the personification of the nation,” arguing that it was a common strategy used by the Swedish Institute in its promotional campaigns. Putting an individual or a fictional character at the center of a narrative to indirectly convey information to the viewer had long been a pedagogical tool in educational cinema, internationally, and in Sweden (Kuhn 1988, 51–56; Schaefer 1999, 30–32). In the Swedish governmental short *Vi ska ha barn!* (We are having children!, Holmsen 1948), for instance, information about the welfare society’s support for expectant parents is communicated as the viewer follows different characters in their encounters with social workers and medical doctors. There are also examples of this structure in films made after *The face of* *Sweden*, which indicates its enduring character. For instance, the educational short film *Barnet* (The child, Cronsioe 1967), based on Nilsson’s images, follows a couple through their visits to the maternity clinic, informs the viewer about various forms of support offered by society, and shows the birth of their baby (see Björklund 2012, 285–286).

**Fig. 1:**
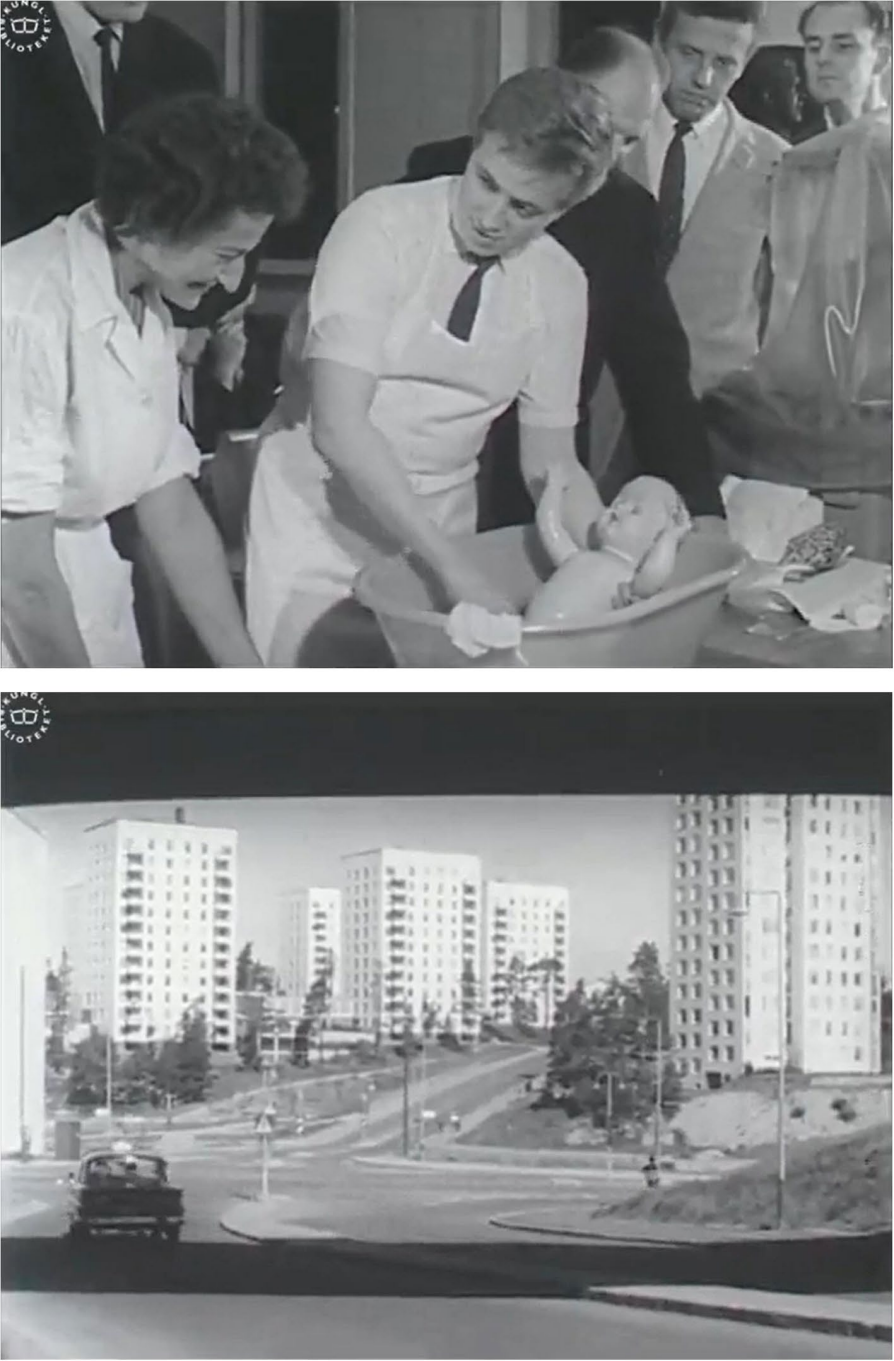
Screenshots from *The face of Sweden: A series of self-portraits*, part 1: “The secure society” (The Swedish Institute/The Swedish Broadcasting Corporation/NETRC 1962), showing the father-to-be bathing a baby doll and the couple traveling to a modern suburb at the end of the film. Source: National Library of Sweden

Through the stories of the two women featured in *The face of* *Sweden*, the film clearly constructs an image of Sweden as a modern country. Part of this is a focus on gender equality, which was an aspect of Swedish society often used to promote Sweden abroad during this period (Glover et al. 2009). For example, one of the women is shown working as a teacher, both at the beginning of the film, when she does not yet know of her pregnancy, and at the end, when she is heavily pregnant. There is also an emphasis on the involvement of her husband in the events depicted. A few scenes show classes intended for fathers-to-be, in which one father is practicing by bathing a baby doll. At the end of the film, he is also present at the delivery, which was an emerging but still unusual practice in the early 1960s (Larsson 2022, 198–199). The film also highlights images of modern hospital care. Long shots of the large Karolinska Hospital in Stockholm are repeatedly shown, and when following the woman with a heart problem, the camera lingers on the advanced technical equipment used to monitor her status. Finally, the episode strongly emphasizes modern housing. In one part, the camera shows several newly built apartment buildings from different angles, emphasizing their number and height by panning and tilting. At the end of the film, we also see the young couple happily traveling by taxi to a modern suburb with their newborn son. This final car scene connotes movement and progression, which are emphasized when the last scene shows a commuter train while the credits start to roll (Fig. 1). The framework of pregnancy and birth also has this function within the larger structure of the film. While parts of the program deal with issues other than maternity care, ending the film with the birth of a baby creates a closure that points to the future. In the scene just before the birth, the voice-over summarizes: “So safeguarded, the hope is that a Swede entering the world today, whatever his background or the circumstances of his birth, will stand a good chance of making the most of the human and material opportunities offered by life itself.”

At the same time, modernity is clearly combined with a notion of the natural. This is explicitly articulated by the voice-over when he explains that the childbirth preparation classes are intended to prepare a pregnant woman for “natural delivery, which she can experience fearlessly, without recourse to pain-killing drugs or anesthetics.” This language clearly evokes the method of Grantly Dick-Read, disseminated through his books *Natural childbirth* from 1933 and *Revelation of childbirth* from 1941, both published in Swedish in the 1950s, although his name is not mentioned in the film. Dick-Read’s method began being practiced in Swedish hospitals in the early 1950s, and maternal preparation classes soon developed into a large movement (Jansson 2008, 68–74; Larsson 2022, 169–173). While there was skepticism about the method even in the 1950s and it did not influence obstetric care to any great extent (Jansson 2008, 72–74; Larsson 2022, 172), the speaker in *The face of Sweden* states that it had been used with “indisputable success” in Sweden for more than ten years.

**Fig. 2:**
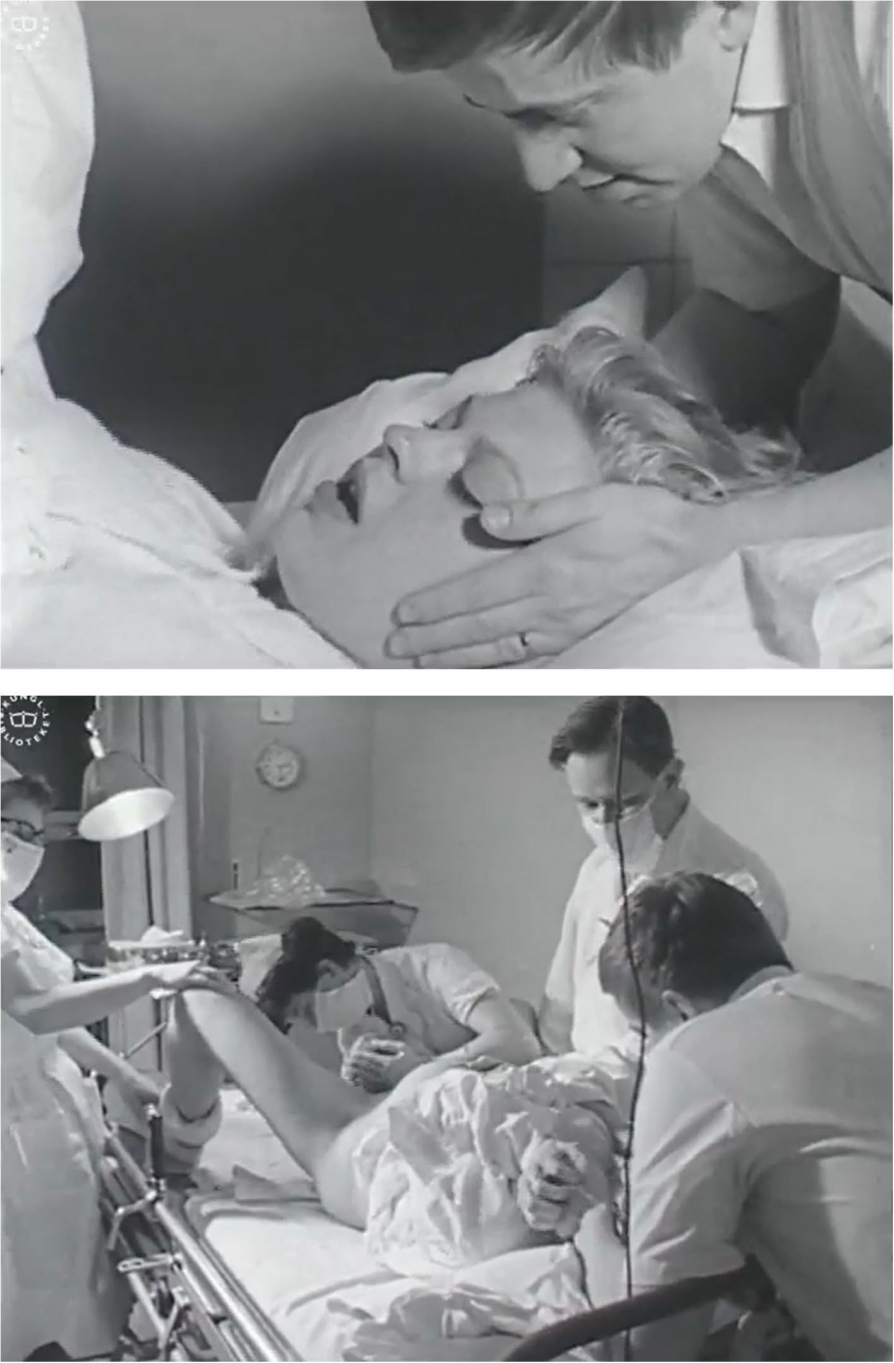
Screenshots from the birth scene in *The face of Sweden: A series of self-portraits*, part 1: “The secure society” (The Swedish Institute/The Swedish Broadcasting Corporation/NETRC 1962) showing the face of the woman in labor and the last stage of the delivery when the baby is born. Source: National Library of Sweden

The childbirth scene at the end of the film also shows a “natural birth.” The scene is quite short and gives the impression of a quick and uncomplicated delivery. It begins with a shot of the woman lying in a hospital bed in the supine position, with the camera focusing on her face. Her husband is standing behind her, comforting her by holding his hands around her head. The camera then alternates through movements and cuts between showing shots of a doctor and two nurses at the end of the bed by the woman’s spread legs and shots showing close-ups of the woman’s strained face as she pushes the baby out. She makes sounds of pain during the scene, but she does not scream. When the baby arrives, the camera is positioned at a greater distance, behind the shoulder of the birthing woman, which can be interpreted as being her perspective. After this, a few shots show the relieved faces of the woman and her husband, and one of the nurses holding up the baby to show the parents (Fig. 2). While still within the bounds of what Swedish censorship would likely have accepted in films of childbirth in previous decades as well—as it showed a vaginal birth—this scene nevertheless constructs a very different picture of birth compared with that conveyed by many theatrically distributed sex education films in earlier decades. In these films, childbirth scenes usually displayed the moment when the baby emerged from the vagina, omitting any shot of the woman’s face, and the camera was often placed between her spread legs and aimed at the vaginal canal.

While differing in how it portrayed birth, *The face of* *Sweden* was in many ways typical of how reproduction was represented and how information about welfare state support was communicated through moving images in Sweden during this era. At the same time, media discussion of it also allowed a different perspective to appear. The same week as the program was to be shown in Sweden, *Röster i radio-tv* published a long article written by Birgitta Hedlund, the woman who gave birth on camera in the program. Hedlund described becoming a mother as a “violent change” and expressed her conflicting feelings about it, which included disappointment, exhaustion, and loneliness, and she criticized the romanticized and one-sided images of happy motherhood spread in books and the media:

Good advice is chanted monotonously and motherly, and you know that there are no other alternatives. Besides, you are happy with your child. Overjoyed? Yes and no, happiness is, thank goodness, nuanced. But which mother dares to say that she is both disappointed and deeply tired of everything sometimes? That she is both lonely, and afraid of remaining that way? That her life will forever be about being subordinated to her child and that she feels unhappy because of it! (Hedlund 1963, 15^3^)

Although the program itself did not devote much time to the views of the women portrayed, there was still openness in the wider media culture to give expression to these experiences.

## Feminist criticism of medicine and childbirth as a crisis

These types of experiences and criticism, however, would come to the forefront much more clearly in the 1970s. At that time, a great deal had changed in both obstetric care and society at large. In the Western world in general, the 1960s saw new ideals emerging in obstetrics, in which “active management of labor” gradually replaced the older notion of obstetrics as the “art of waiting.” This entailed an increasing emphasis on methods such as the Cesarean section and induced labor, and in Sweden, it was also paralleled by the centralization and rationalization of hospital care, as well as by closer collaboration among engineers, medical doctors, and industry. New technologies for monitoring and controlling labor also appeared, such as cardiotocography (CTG), and new methods of providing pain relief during delivery (Jansson 2008, 75–83; Larsson 2022, 166–168, 173–181).

The spread of information about pregnancy and childbirth also increased during this period, and its character was changing. In 1968, the publisher Bonnier, for instance, started *Vi föräldrar* (We parents), a colorful magazine targeting parents and parents-to-be. Commercial companies such as manufacturers of diapers and baby food had also begun publishing informational brochures and books, which were distributed to new or expectant parents through maternity clinics, child health centers, or direct mail. In contrast to much of the information distributed by governmental bodies, these publications put great emphasis on layout and often included many color photographs and headings in large print, often set in bold. In the late 1970s, a public inquiry noted that commercial material of this type likely constituted a substantial part of the information available to parents on pregnancy and childbirth (Barnomsorgsgruppen 1978, 131).

At the same time, with the rise of the New Left and second-wave feminism, critical perspectives on medicine, reproduction, and the welfare state gained currency. By the end of the 1960s, pain relief during labor became the object of considerable debate. The new women’s movement was very engaged in this issue, but its perspective on the matter shifted. In the early 1970s, women’s right to pain relief was considered a crucial demand, but many changed their views in the late 1970s, instead promoting the view that women should reclaim power over their bodies so that they could give birth “naturally” (Jansson 2008, 149–191, 235–263). At this time, Dick-Read’s method had fallen out of fashion and instead Lamaze’s psychoprophylaxis—a method originating in the Soviet Union, where techniques of controlled breathing and relaxation were used to relieve pain—became increasingly influential (Jansson 2008, 215–231; Larsson 2022, 206, 216–230; see also Michaels 2014). A critical stance towards medicine and reproductive politics can also be noted in various cultural expressions of this era (Björklund 2019a, b; Isaksson 2007, 200–204; Jansson 2008, 236–238; Larsson 2006, 125–167).

An orientation towards social criticism and left-wing perspectives more generally can also be seen clearly in Swedish television coverage. Critique of the Swedish welfare state had previously been voiced, but the late 1960s was the era when the press, radio, and television developed into media in which taking a critical stance on society was prominent (Vesterlund 2020, 131–133). In the late 1960s and 1970s, a radical documentary film movement gained a strong platform in television (Furhammar 2013; Nilsson 1989; Wahlberg 2017). Moreover, Swedish television drama of this era was characterized by realism and social commitment, often portraying life in the Swedish welfare state as “an existence characterized by displeasure and despair” (Vesterlund 2020, 129). Healthcare was a prominent issue in these television productions, in which “the system” was often identified as the root of the problems (Vesterlund 2020, 135–138).

The first episodes of *Livskris* and *Omställningen* are clear expressions of these developments and seem in certain ways to embrace a completely opposite view of Swedish society from that of *The face of* *Sweden* and other educational programs on pregnancy and birth from this era. One main difference lies in their production histories: while *The face of* *Sweden* was the product of a governmental agency concerned with the image of Sweden abroad, both *Livskris* and *Omställningen* came about on the initiative of women filmmakers and television producers who developed their ideas based on professional or personal experiences. Monica Kempe, the main producer of *Livskris*, conceived of the series based on having worked for ten years as a psychologist and social worker, while Maj Wechselmann’s own experiences of having her first child sparked the idea behind *Omställningen* (Mannberg 1973, 17; Ljunggren 1974). The programs also differ stylistically from previous programs. Although they both use voice-over narration to directly address viewers, they also make use of strategies employed in the documentary tradition of *cinéma vérité*, in which the filmmaker interacts with the surrounding world through interviews, often with ordinary people, in this case pregnant women or women who have recently had a child. The speakers in both programs are also women, which is a clear deviation from the norm of the authoritarian male voice-over used in most previous examples.

Women’s own voices and experiences are central in these films. This esthetic was recurrent in early feminist documentaries of the 1960s and 1970s and can be related to broader feminist aims. For instance, in the widely influential book *Our bodies, ourselves*, published by the Boston Women’s Health Book Collective in the early 1970s, knowledge derived from experience—the notion of “experiential knowledge”—was privileged over the prevailing medical or biological knowledge (Kline 2010). The programs also share a focus on emotions that goes beyond the feminist movement. Historians have shown how “the long 1970s”—that is, the period from the late 1960s to the early 1980s—were characterized by a reorientation of Swedish society towards a strong focus on emotions more broadly, not only within the alternative movements (Bergman et al. 2017; Tillema 2021). In the two television programs explored here, this is evident through their focus on psychological experiences of being pregnant and having a child.

An obvious similarity between the programs can be found in their titles. Both *Livskris* (Life crisis) and *Omställningen* (The change) indicate that pregnancy and having one’s first child are life-changing experiences. This is the major theme of both programs, and many of the interviewed women attested to having experienced an awakening or turning point when they discovered they were pregnant. Both titles also indicate that pregnancy does not only concern the body. This perspective was becoming increasingly prominent in discussions of pregnancy and childbirth in general in the 1970s (Jansson 2008, 193–203). In the case of *Livskris*, however, it was also tied to a more specific discussion of crises in life. *Livskris* was a series of five programs focusing on different types of crises: losing one’s job, undergoing a divorce, experiencing loss, etc. Before the premiere, a longer article in *Röster i radio-tv* reported on the new series. Here, it was presented as being about events that affected people’s entire life situation. This, however, was clearly described in negative terms: “An experience of your entire existence cracking up. Nothing that you know or can do helps you any longer. Your feeling of life itself is hurt” (Mannberg 1973, 17). The producer Monica Kempe argued that the events covered in the programs should be regarded as normal parts of life and not as illnesses. Instead of psychiatric care, she argued that people needed to talk more about feelings when they experienced life crises, but that there were major taboos surrounding this. Kempe also explained that no experts or authorities were part of the program series, because the true experts on these issues were the people who had themselves experienced them (Mannberg 1973, 17).

It seems very likely, however, that experts did influence the program. Kristofer Hansson has shown that “crisis” as a concept was given a new meaning in Swedish psychiatry during this period, influenced by American psychoanalysis. In this new understanding, crisis was seen as a normal part of life and not connected to psychiatric illness. Instead, a crisis was considered something that could happen to anyone, which also had the potential to be an important part of the individual’s psychological development. In Sweden, this new meaning was developed primarily by psychiatrist Johan Cullberg and resulted in new clinical methods. In several clinical trials, crisis psychotherapy was introduced as a new type of treatment offered by outpatient care units at psychiatric clinics (Hansson 2012, 2013, 2018). This was not unknown to the producers of *Livskris*. In another article published before broadcasting the third program in the series, the journalist had met the “crisis team” at Karolinska Hospital, established just over a year earlier, to which Cullberg was a consultant (Mannberg et al. 1973).

In *Livskris*, women express a wide range of emotions about pregnancy and motherhood. Here, joy and happiness are part of some women’s stories, but most of the accounts give expression to negative feelings such as anxiety, disappointment, loneliness, and jealousy. Many of the interviewed women attest to having undergone a transformation when they found out they were pregnant or after the child was born. One woman describes her emotions as “unknown” and “unreal,” and another talks about how having one’s first child means taking a step from being a child to becoming an adult. Another woman, who is still pregnant, describes feeling that she is about to die; she suffers from loneliness and is afraid of the delivery. She furthermore states that she has not been able to talk to anyone about her feelings, neither people at the maternity clinic nor other pregnant women.

The focus on psychology is clearly expressed by the voice-over, who argues that the psychological problems many women experience cannot be reduced to hormonal changes in the body. Instead, these feelings can be explained by women’s situation in modern society. While images of a physical education class for pregnant women are being shown, the speaker says:

Expecting and giving birth to a child means you are occupied by a course of events beyond your control. You surrender to primitive forces you cannot control and that are in opposition to the objective and rational way of life we are used to living and for which society is organized. We expect to see pregnancy and childbirth primarily as a physical event. But most women feel, more or less consciously, that it concerns their entire life.

Here, images of pregnant women exercising serve to illustrate society’s focus on the physical aspects of pregnancy, rather than—as in *The face of Sweden*—presenting a positive image of Swedish society’s focus on maternal health.

**Fig. 3:**
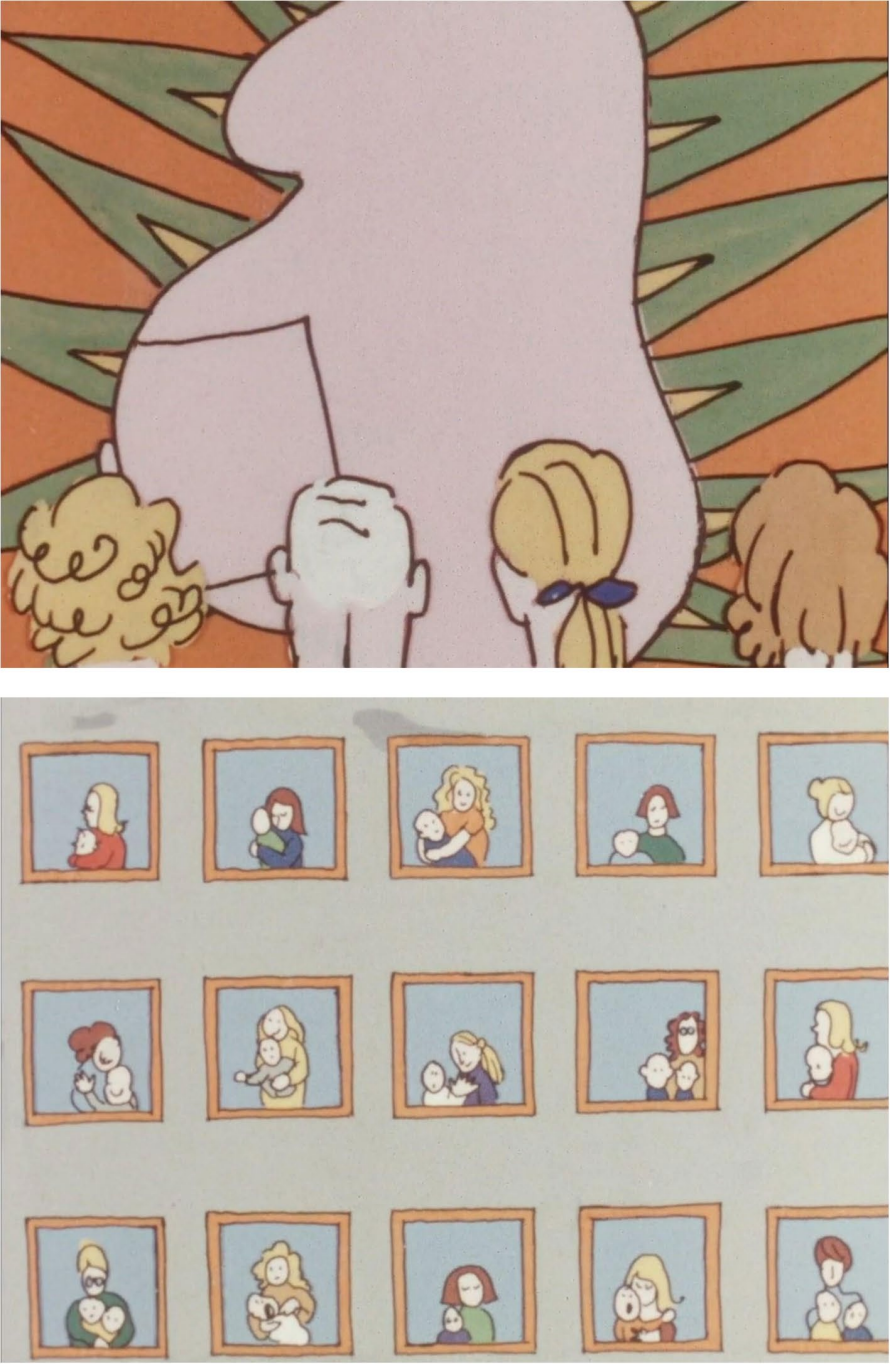
Screenshots from two animated scenes in *Omställningen* (Wechselmann 1974), depicting an anxiety-laden dream during pregnancy and a number of women and their children isolated from one another in an apartment building. Source: https://www.majwechselmann.se#omkvinnor

The psychological perspective on pregnancy is also clear in *Omställningen*. This film has a different tone from that of *Livskris*, as it uses humor to a greater degree to convey its argument. The women interviewed in this film also laugh more while talking, and none of them expresses the same kind of deep anxiety as do some of the women in *Livskris*. The film is also more overtly political. Here, voice-overs, intertitles, interviews, and animated sequences are used to convey an explicit Marxist and feminist argument about how capitalist society affects women’s experiences of motherhood. Central to the film is a criticism of the overflow of information distributed to expectant parents by commercial companies, such as producers of diapers and baby food, and it is often pointed out how the images of pregnancy and of having children conveyed in this material are idealized, romanticized, and far from the unequal reality faced by Swedish mothers. Nevertheless, the film’s emphasis on difficult experiences of becoming a mother is similar to that in *Livskris*. For example, in one part discussing pregnancy, the focus is not on what happens in the body, but instead on different anxiety-laden dreams pregnant women sometimes have, dreams that are illustrated using animation (Fig. 3). This is continued in another animation that explicitly connects depression during pregnancy to women’s roles in society. Here, the camera moves past a number of doors labeled with different words—“dance,” “politics,” “the union,” “career,” “schools,” and “hobbies.” After a cut, all these doors are shut and combined into a single door with the word “motherhood” above it. Here, as in *Livskris*, the depression that women may experience during pregnancy is explained by society’s limiting gender roles.

The focus on psychology rather than biology is also connected to another central issue in the programs: criticism of Swedish maternity and obstetric care. In *Livskris*, this is explicitly expressed in a sequence from a maternity clinic. Here, several women are shown as they are being weighed and examined. On the soundtrack, women’s voices can be heard testifying to negative experiences of visiting these clinics. After this, the voice-over says:

The maternity clinic is the societal institution that is supposed to give an expecting woman the help she needs. But just like the rest of our society today, the focus of preventive maternity care is technical. The mother-to-be is treated like a machine for production. You weigh, measure, take tests. [….] The pregnant woman is left behind with her individual emotional problems. In this way helping communicate to her the idea that the matter entirely concerns her body, which makes her feel abnormal when she reacts with new and strange feelings.

This is also clear in *Omställningen*. For example, in some scenes, hospitals are compared to factories and constructed as impersonal and sterile environments in which children are “produced.” This is tied to a historical analysis presented in the film according to which modern industrial society is contrasted with an older agricultural society. While women in the past lived in large households where they could teach one another, it is said, women today are isolated from one another in newly built apartment complexes in the suburbs, implying that the sharing of experiences has decreased in favor of written information produced by industry. By the early 1970s, the construction of new suburban dwellings had increased dramatically. In 1965, the Swedish Parliament had decided on an enormous building project—the “Million Program”—through which a million homes would be constructed in ten years. From the end of the 1960s onward, however, this development was increasingly criticized (Gråbacke et al. 2010; Hall 1999), as clearly exemplified by *Omställningen*. In one sequence, an animated scene shows a number of windows in a building, with a woman’s head and one or two children visible in each window, while the speaker says: “Here we can sit all day in our own little cell and get a patent on our child” (Fig. 3). This is followed by a row of shots showing a number of suburban areas with similar architecture. This was clearly in line with other feminist representations of the time. For example, in filmmaker Mai Zetterling’s *Flickorna* (*The girls*, 1968), modernist buildings designed by British architect Ralph Erskine were part of an implicit criticism of the rational welfare society (Larsson 2007, 151–152). Another example is Anna Sjödahl’s painting *Vår i Hallonbergen* (Spring in Hallonbergen) from 1972. Here, the figure in Edvard Munch’s famous painting *The Scream* (1893) is placed in an apartment with a small child on the floor in front of a window showing a modernist high-rise building in the background. Hallonbergen—a Stockholm suburb built as part of the “Million Program”—was also one of the areas featured in *Omställningen*. Here, one sees a view of healthcare, modernity, and housing completely opposite to the one constructed in *The face of* *Sweden*.

Both programs also visualize childbirth. In *Livskris*, this is done in quite a short scene at the beginning of the program, without much context or drama. Directly after the title sequence, a woman is shown in close-up in a bed breathing nitrous oxide from a mask. Then, from a distance, the camera shows her lying on her back in a hospital bed, with a midwife and a doctor by her side. After a few seconds, and a short close-up of the doctor, the camera cuts to a shot of the baby having appeared between the woman’s legs, followed by a shot of the woman’s smiling face as she hears the baby’s scream. The short duration of the scene and its placement at the beginning of the program function to decenter the physical aspects of birth in the program, making it different from many educational films in which birth is positioned as the climactic ending of a film. In *Omställningen*, the birth scene also differs from dominant representations, which was an explicit aim of the program. In an interview in 1974, Wechselmann said:

Take something like childbirth, for instance. Several films have been made about that. But how are they shot? Well, you see the woman’s bottom and the child coming out. It is mostly men who’ve made these films. And in some way, that says something about how far away they are from the experience. A bottom is not very interesting, we all look approximately the same. In our program, I’ve filmed the face of the woman instead. Her real-life face, which expresses how she feels up to the birth of the child. (Ljunggren 1974, 5)

**Fig. 4:**
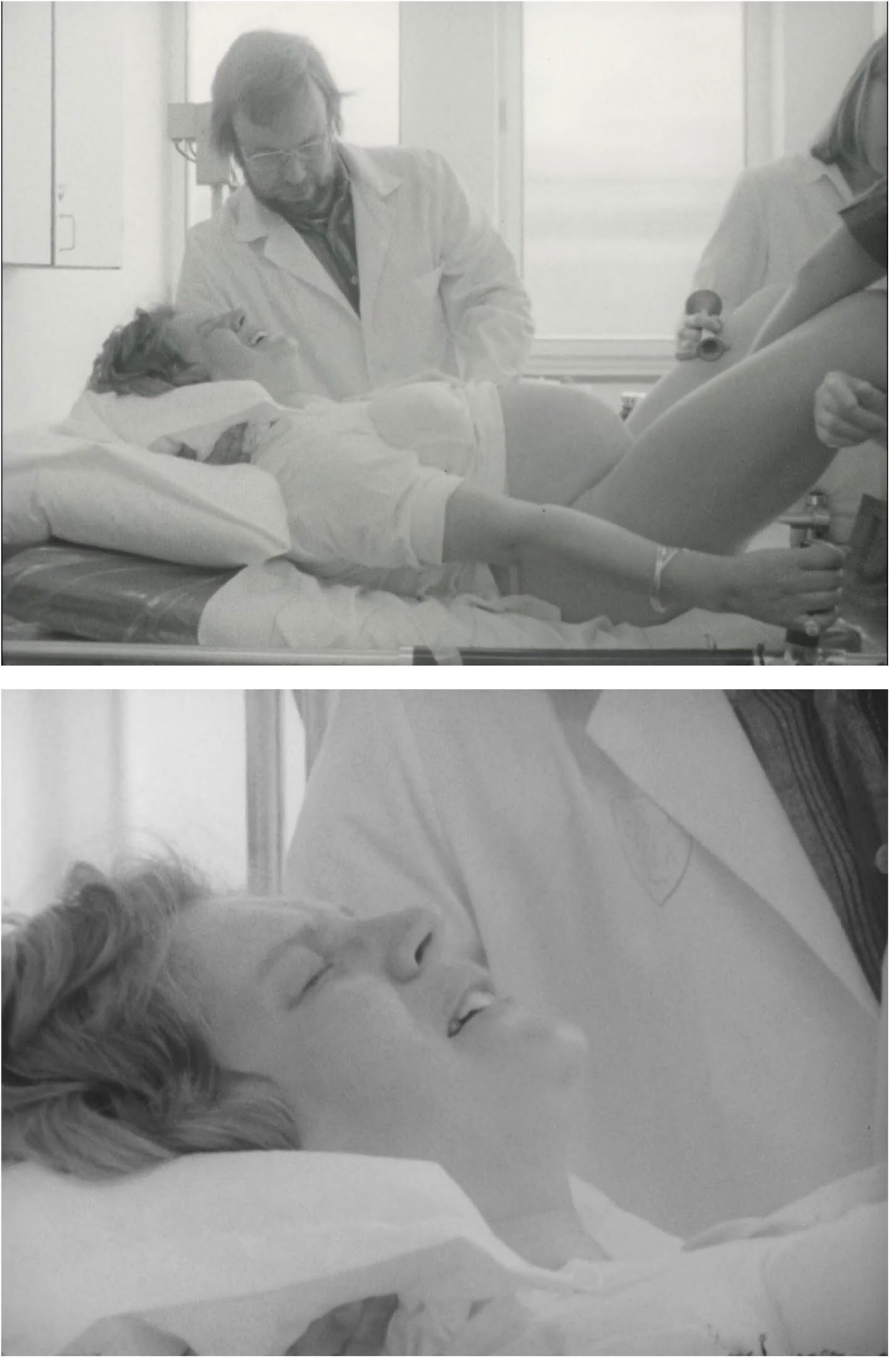
Screenshots from the birth scene in *Omställningen* (Wechselmann 1974), showing the woman in labor filmed from the side and a close-up of her face. Source: https://www.majwechselmann.se#omkvinnor

In *Omställningen*, the woman giving birth is followed through all the phases of labor, from early contractions to the delivery, with ellipses compressing the duration of the events. The film even shows the birthing of the placenta, which is very unusual. When the woman pushes the baby out, the camera is not positioned in what is understood to be the doctor’s or midwife’s perspective, that is, between the spread legs of the woman and directed at the vaginal opening. Instead, the woman is filmed from the side, so that most of her body is visible, and the shots alternate between showing her full body and showing close-ups of her face, her husband, and the medical personnel (Fig. 4). While it is true that this way of framing birth differed from many previous representations, it was, as I have shown, not unique—the depiction in *The face of* *Sweden* was quite similar and there are other examples as well. Just as in *The face of Sweden*, the film also intends to show a woman giving birth without medical interventions, but here, psychoprophylaxis is used instead of Dick-Read’s method.

## Conclusions

In this article, I have analyzed how the representation of childbirth changed in Swedish public service television from the early 1960s to the early 1970s. My focus has been limited to aspects related to the production of these works and to their textual meaning-making, while audience responses to them and their wider social impact remain to be studied. From the examples I have highlighted, a change can clearly be noted from an educational mode to a critical feminist one, in which the relationships to the welfare state seem almost the opposite to one another. In *The face of* *Sweden*, childbirth was treated as a natural event, although one for which women needed to prepare. Society was constructed here as a crucial factor for maternal health, and the Swedish welfare state—with its antenatal preparation classes, advanced hospital care, and modern housing—was constructed as safeguarding the future life of its citizens. Only a decade later, in *Livskris* and *Omställningen*, childbirth was reframed within a criticism of exactly these aspects of Swedish society. Maternity care was seen as technical, hospitals as industrial, and modern suburbs as isolating—factors, it was argued, that contributed to women experiencing pregnancy and childbirth as a crisis. This development might seem expected, given the larger changes in society during the period, but it is not straightforward. For example, the ideal of natural birth is clearly part of both eras. While new medical technologies such as CTG and medical pain relief developed in the 1960s were indeed the objects of considerable attention in several television programs other than those discussed here, it is also noteworthy that vaginal births clearly predominate in all programs from the period. Just as today, the widespread division between the “medical model” and the “natural model” of birth was clearly a subject of discussion during this period, but *The face of* *Sweden* shows that a connection between Swedish modernity and the medicalization of birth is not self-evident. Rather, the “natural” was also made part of the image of Sweden.

Moreover, the shift I describe is not clear-cut, because the educational format had not disappeared from television or other media in the 1970s. As mentioned, in school films of the period, a biological focus on reproduction was dominant well into the 1970s. Moreover, commercial companies were an important producer of information for expectant parents. The producers of *Livskris* and *Omställningen* saw themselves as creating an alternative to these dominant representations. Yet, even though several other critical examples also existed, the larger visual culture around birth still featured many depictions of pregnancy and childbirth that were more in line with a traditional educational mode of representation. Representations of birth during these years were conveyed in a variety of different media produced and used by many different actors, and there was widespread circulation of images among different media featuring competing forms of representation.

Nevertheless, the importance of the fact that *Livskris* and *Omställningen* were given space in the medium of television should not be understated—television arguably being the most influential medium of the time. It is possible that the focus on women and the family in early Swedish television culture was a stepping stone for the more critical expressions that followed. Kleberg argued that, while many of the programs produced by the unit working on the home and family express a view of women in which diligence and good behavior are central, they may also have contributed to bringing issues such as women’s labor and everyday life into focus, helping pave the way for discussions of these issues within the women’s movement (Kleberg 1999). Similarly, the openness around birth in films such as *The face of Sweden*, its focus on gender equality, and the space given to Birgitta Hedlund to express her feelings in *Röster i radio-tv* were part of a more general focus on these issues within the new public space of television, which might have made it easier to produce more critical programs on these issues later. Compared with our present time, a final remark is that, even though people’s access to films of childbirth was more limited during this period than it is today, depictions of birth were indeed to be found in many different media and contexts. Moreover, the dominance of television as a visual medium in the 1960s and 1970s and the specific way in which its character developed in Sweden meant that representations understood as radical or oppositional had much wider national distribution than is possible to achieve through either television or social media today.
